# Towards Context-Rich Automated Biodiversity Assessments: Deriving AI-Powered Insights from Camera Trap Data

**DOI:** 10.3390/s24248122

**Published:** 2024-12-19

**Authors:** Paul Fergus, Carl Chalmers, Naomi Matthews, Stuart Nixon, André Burger, Oliver Hartley, Chris Sutherland, Xavier Lambin, Steven Longmore, Serge Wich

**Affiliations:** 1 School of Computer Science and Mathematics, Liverpool John Moores University, James Parsons Building, Byrom Street, Liverpool L3 3AF, UK; c.chalmers@ljmu.ac.uk; 2 Chester Zoo, Upton-by-Chester, Chester CH2 IEU, UK; n.matthews@chesterzoo.org (N.M.); s.nixon@chesterzoo.org (S.N.); 3 Welgevonden Game Reserve, P.O. Box 433, Vaalwater 0530, South Africa; andre@welgevonden.org; 4 School of Mathematics and Statistics, Mathematical Institute, University of St Andrews, North Haugh, St Andrews KY16 9SS, UK; oh57@st-andrews.ac.uk (O.H.); css6@st-andrews.ac.uk (C.S.); 5 School of Biological Sciences, University of Aberdeen, Tillydrone Avenue, Aberdeen AB24 2TZ, UK; x.lambin@abdn.ac.uk; 6 Astrophysics Research Institute, Liverpool John Moores University, IC2, Liverpool Science Park, 146 Brownlow Hill, Liverpool L3 5RF, UK; s.n.longmore@ljmu.ac.uk; 7 School of Biological and Environmental Sciences, Liverpool John Moores University, James Parsons Building, Byrom Street, Liverpool L3 3AF, UK; s.a.wich@ljmu.ac.uk

**Keywords:** wildlife conservation, deep learning, object detection, large language models, vision transformers, biodiversity monitoring

## Abstract

Camera traps offer enormous new opportunities in ecological studies, but current automated image analysis methods often lack the contextual richness needed to support impactful conservation outcomes. Integrating vision–language models into these workflows could address this gap by providing enhanced contextual understanding and enabling advanced queries across temporal and spatial dimensions. Here, we present an integrated approach that combines deep learning-based vision and language models to improve ecological reporting using data from camera traps. We introduce a two-stage system: YOLOv10-X to localise and classify species (mammals and birds) within images and a Phi-3.5-vision-instruct model to read YOLOv10-X bounding box labels to identify species, overcoming its limitation with hard-to-classify objects in images. Additionally, Phi-3.5 detects broader variables, such as vegetation type and time of day, providing rich ecological and environmental context to YOLO’s species detection output. When combined, this output is processed by the model’s natural language system to answer complex queries, and retrieval-augmented generation (RAG) is employed to enrich responses with external information, like species weight and IUCN status (information that cannot be obtained through direct visual analysis). Combined, this information is used to automatically generate structured reports, providing biodiversity stakeholders with deeper insights into, for example, species abundance, distribution, animal behaviour, and habitat selection. Our approach delivers contextually rich narratives that aid in wildlife management decisions. By providing contextually rich insights, our approach not only reduces manual effort but also supports timely decision making in conservation, potentially shifting efforts from reactive to proactive.

## 1. Introduction

Camera traps have proven useful in wildlife conservation efforts, offering insights into animal population dynamics and habitat use across large geographical areas without the need for direct human observation [1,2]. Through image and video data collection, these motion-triggered devices provide novel information, often inaccessible by other means, for biodiversity assessment and the evaluation of conservation strategies [3]. However, the sheer volume of data being generated by camera trap projects presents unique challenges [4]; environmental factors, such as moving vegetation or changing light, often lead to false positives [5], adding noise that complicates species identification and demands additional resources to manage [6,7].

Combined, these challenges make processing and analysing the datasets particularly complex [8]. Traditionally, the initial stages of camera trap image analysis involve manual annotation, often conducted by experts or through citizen science initiatives [9,10]. This annotation process, which converts images into a structured format, such as CSV for further analysis, is time-consuming [11], costly, and prone to human error [12]. Subsequent analysis typically requires additional software tools, such as R [13], and a deeper understanding of statistical and special methodologies to derive meaningful insights.

In relation to manual annotation limitations, automated detection models, such as MegaDetector [14], have been developed to assist in identifying general categories like animals, humans, and vehicles in images [15]. By reducing the need for manual review, MegaDetector significantly helps to improve workflow efficiency, making it an essential tool in ecological studies [16,17]. However, while effective at broad categorisation, MegaDetector lacks species-specific identification, limiting its utility in detailed ecological assessments [18,19].

To address this issue, advances in object detection models [20], such as YOLO (You Only Look Once), have introduced improved capabilities for species-specific identification [21,22], making them increasingly applicable to camera trap data [8,23]. As detailed in this paper, YOLO is highly effective at detecting and classifying different species in challenging environments captured in low-quality images. Constrained by the architecture of the model and its tailored training set, it is, however, unable to detect anything outside of what it has been trained on. This is a significant limitation in situations where context-rich information is needed [18], such as animal behaviours (e.g., sitting, standing, feeding) and environmental context (e.g., habitat damage [24] or the presence of invasive plants) [23].

Recognising the critical role of contextual understanding in enhancing detection accuracy, recent research has increasingly focused on multimodal large language models (MLLMs) [25], such as ContextDET, which integrates cues from human–AI interactions to improve object detection in complex scenes [26]. ContextDET, a vision language model (VLM) [27] utilises a generate-then-detect framework, combining visual encoders, pre-trained language models, and visual decoders to locate and identify objects within diverse contextual environments, effectively responding to open-ended queries [28]. Building on these advancements, models like VCoder introduced versatile vision encoders, designed specifically to enhance object perception tasks, such as counting or identifying entities within cluttered scenes, where traditional MLLMs may struggle [29]. Meanwhile, VisionLLM offers a flexible, open-ended task approach by aligning language instructions with vision tasks, which enables a range of vision-centric functions like object detection, image captioning, and visual reasoning [30]. Although these frameworks have shown success in urban applications, their adaptation for conservation remains limited, presenting a valuable opportunity to leverage their contextual capabilities in complex wildlife monitoring environments [31].

In response to this need, we propose an integrated approach that combines deep learning-based vision and language models to enhance camera trap image understanding [32]. Specifically, our method merges the object detection capabilities of YOLOv10-X [33] with the vision–language understanding of Microsoft’s Phi-3.5-vision-instruct transformer model [34,35]. In addition, our system integrates a retrieval-augmented generation (RAG) framework [36], allowing it to draw on external sources, such as the IUCN Red List [37], for answering complex queries about camera trap images. While existing tools, such as the R package “traitbase” [38] and platforms like the Open Traits Network [39], offer the ability to attach additional trait characteristics (e.g., average weight, IUCN status) to species lists, they often require users to have programming expertise and domain knowledge. In contrast, our system automates this process, allowing users to obtain insights through natural language queries, such as “What species is in this image, how much does an average adult weigh, and what is its IUCN status?”—information that cannot be obtained through direct visual analysis. By integrating these capabilities, our approach aims to simplify workflows and provide faster access to actionable data, making advanced tools accessible to a broader audience, including non-specialist users.

Building on prior detection and contextual models [21,22], our approach generates structured, context-aware outputs that consolidate species-specific and environmental insights from camera trap data. While these outputs provide foundational information, such as species identification, estimated counts, and behavioural or habitat context, further analysis is still necessary to interpret these insights for specific stakeholder needs. This could include translating the data into biodiversity metrics, such as biocredit trading [40], or monitoring environmental changes, like habitat destruction or poaching activities (for example through weapon detection) [30,41]. By streamlining the annotation process and providing immediate insights, our approach has the potential to enhance conservation efforts and resource allocation [42,43]. However, we acknowledge that the full impact of this system will depend on integrating its outputs into broader conservation workflows and decision-making processes.

The remainder of this paper expands on this approach. Section 2 details our methodology, presenting the innovative solution we developed. Section 3 presents the results, followed by a discussion in Section 4, with final conclusions and future directions in Section 5.

## 2. Methodology

Our system integrates object detection, vision–language modelling, and retrieval-augmented generation (RAG) to deliver detailed, context-rich reports about wildlife in natural habitats. By combining these complementary components, our approach addresses the limitations with traditional camera trap methods, offering a more comprehensive and automated tool for environmental monitoring. The methodology consists of four broadly defined stages: object detection for animal identification, vision–language modelling for visual understanding and image querying, and RAG for contextual enrichment using external knowledge.

Below, we provide a system overview before outlining each stage in detail, describing the specific configurations and processes used to achieve our objectives.

### 2.1. System Overview

**Figure 1:**
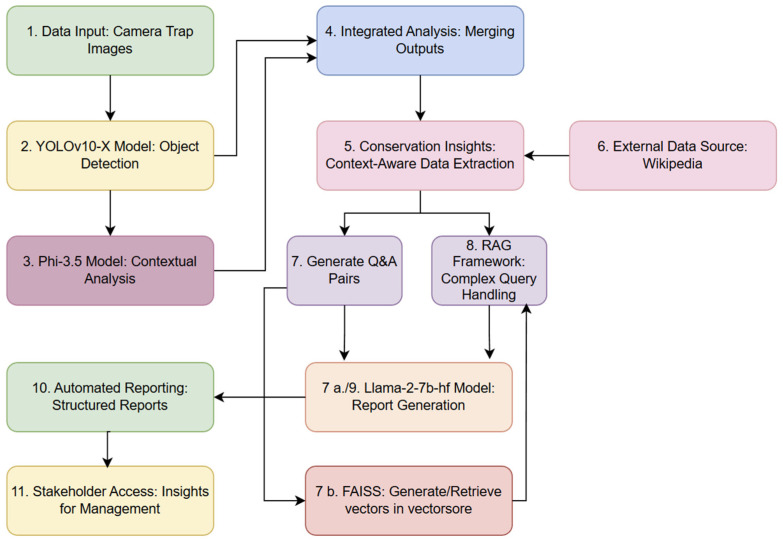
Flow chart illustrating an overview of the workflow for the YOLOv10-X and Phi3.5-vision-instruct model integration for context-rich camera trap data processing.

Figure 1 shows the workflow to automate the transformation of camera trap images into actionable insights and structured reports, using object detection, contextual analysis, and RAG. The process begins with the ingestion of camera trap images (Step 1), which are analysed by the YOLOv10-X model to detect and spatially localise animal species (Step 2). Contextual analysis is conducted via the Phi-3.5 model, which reads the bounding box labels generated in Step 2 and extracts domain-specific information from other identified objects. The outputs from these models are then integrated in Step 4, producing a comprehensive dataset optimised for downstream analysis and interpretation.

Conservation-specific insights are extracted with contextual awareness, further enriched by supplementary information retrieved from external sources, such as Wikipedia (Step 5 and 6). This system generates structured question-and-answer pairs, facilitating systematic information retrieval and report generation (Steps 7, 7a, 7b, and 8). Complex queries are addressed through a RAG framework, which leverages this contextual information to deliver precise and relevant responses. Insights are compiled into structured reports using the Llama-2-7b-hf model [44], which formats the outputs for direct accessibility by stakeholders (Step 10 and 11). Additionally, a FAISS-based vector store [45] underpins efficient query management, enabling rapid retrieval and real-time access to critical insights.

### 2.2. Data Collection

By collaborating with global conservation organisations, Conservation AI [21] (an AI platform that provides species-specific object detection models for offline and online camera trap image processing) has compiled diverse camera trap datasets that represent a wide range of habitats. This diversity in species and environments ensures that the AI models developed are robust and adaptable across ecosystems, significantly enhancing their utility for global conservation efforts.

**Figure 2:**
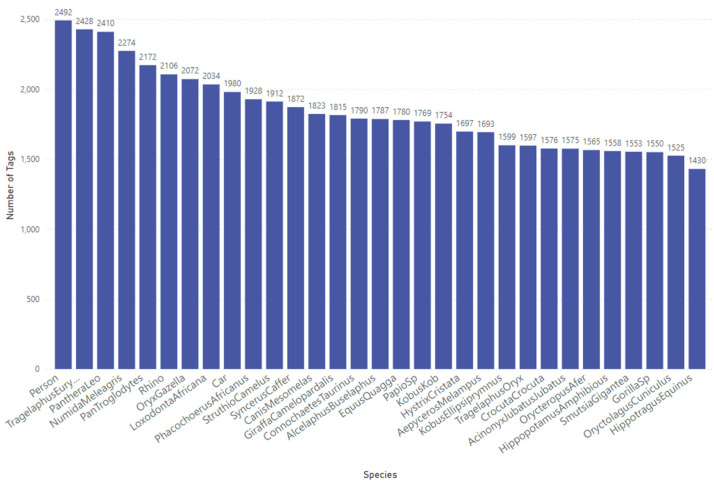
Class distribution for the Sub-Saharan Africa dataset used to train the YOLOv10-X model to localise and detect mammals, birds, people, and cars.

For this study, we utilised our Sub-Saharan Africa dataset, which contains 57,120 tagged objects across 41,111 RGB images representing 31 distinct classes (29 animal species, 1 person, and 1 car), as shown in Figure 2. These camera trap images were sourced from across southern and central African regions. High-quality image tagging, performed by specialists and managed through our in-house quality control process, ensures precise bounding box annotations. This consistency is essential for optimising model performance, given the complexity and scale of the dataset.

The dataset is divided into training, validation, and test sets with an 80:10:10 split. The training set facilitates model learning, while the validation set is used for hyperparameter tuning, and the test set to evaluate the model’s performance (mean average precision (mAP) and intersection over union (IoU)) (see Section 2.8) [46]. This split ensures the model generalises well to unseen data and avoids overfitting (overfitting is when the model becomes too tailored to its training data, sometimes fitting it exactly, which leads to a model that struggles to make accurate predictions or inferences on data outside the training set). Additionally, 602 independent camera trap images were collected to evaluate the trained YOLOv10-X object detection model and the Phi-3.5-vision-instruct model’s ability to identify additional objects outside the capabilities of the YOLOv10-X model.

### 2.3. Object Detection Model

**Figure 3:**
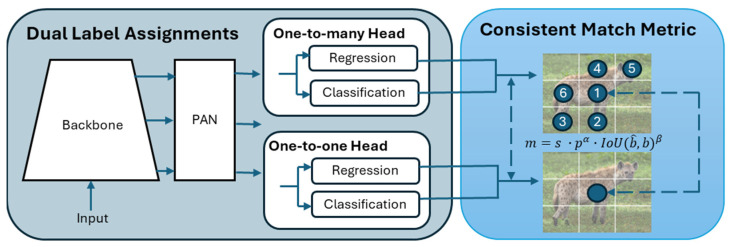
Overview of the YOLOv10 architecture.

The first component in our system is the YOLOv10-X model [33], which is used to detect, classify, and localise animals in camera trap images. The YOLOv10 architecture (Figure 3) incorporates an enhanced version of CSPNet (Cross Stage Partial Network) [47] to improve gradient flow and reduce computational redundancy, making it highly efficient for large-scale datasets. The CSPNet backbone extracts key features from input images, crucial for handling the variability seen in wildlife camera trap imagery. To ensure robustness across conditions, the model’s neck uses path aggregation network (PAN) layers for effective multiscale feature fusion, enabling the detection of animals of various sizes, from small birds to large mammals [48]. During training, the model uses a one-to-many head to generate multiple predictions per object, enhancing learning accuracy. In the inference phase, it shifts to a one-to-one head, eliminating the need for non-maximum suppression (NMS) and reducing latency [49].

YOLOv10X incorporates lightweight classification heads, spatial–channel decoupled downsampling, and rank-guided block design, which reduce computational overhead without compromising accuracy. Large-kernel convolutions [50] and partial self-attention modules [51] further enhance its ability to process complex scenes, without increasing computational cost. These optimisations ensure that YOLOv10-X offers a powerful, efficient solution for wildlife monitoring where speed and accuracy are crucial. The model is deployed on an NVIDIA Triton Inference Server, providing serverless API endpoints for easy integration without requiring extensive infrastructure [52].

### 2.4. Model Training

Using the Microsoft COCO dataset [53], YOLOv10-X was trained using 8 NVIDIA RTX 3090 GPUs over a 10-day period, with additional transfer learning using our Sub-Saharan Africa wildlife dataset. Transfer learning was performed on a Gigabyte server equipped with an AMD EPYC 7252 processor and 128 GB of RAM. To enable accelerated learning, the server utilised 8 Nvidia Quadro A6000 GPUs, offering a total of 384 GB of GDDR6 memory. The training pipeline was implemented using PyTorch 2.0.1 with CUDA 11.8, ensuring efficient hardware utilisation and seamless model training. The key hyperparameters employed during the training process were designed to balance accuracy, efficiency, and generalisation:

- Image size: 640 pixels, chosen to optimise detection accuracy, while maintaining computational efficiency, aligning with the dataset’s mean resolution;
- Batch size: 256, to enable stable weight updates without exceeding GPU memory capacity;
- Epochs: 50, providing sufficient time for convergence while minimising overfitting risks;
- Learning rate: 0.01, enabling balanced gradient updates for steady training progress;
- Momentum: 0.937, enhancing gradient stability and directional convergence during training.

To further improve model generalisation and reduce overfitting, real-time data augmentation was employed. These techniques introduced variability in the training data without increasing the dataset size.

- Hue adjustment (hsv_h = 0.015): randomly modified by up to 1.5%, introducing subtle colour shifts;
- Saturation adjustment (hsv_s = 0.7): altered by up to 70%, diversifying the colour intensity;
- Brightness adjustment (hsv_v = 0.4): adjusted by up to 40%, simulating various lighting conditions;
- Horizontal flip (fliplr = 0.5): applied with a 50% probability, increasing the invariance to directionality;
- Translation (translate = 0.1): randomly shifted up to 10%, enhancing the robustness to positional variations;
- Scaling (scale = 0.5): objects were resized by up to 50%, improving detection across size variations;
- Random erasing (erasing = 0.4): applied to 40% of images, simulating occlusions by randomly removing portions of the image.

The final trained YOLOv10-X model is well-suited for handling challenging real-world imagery, such as low-quality or obscured camera trap images. It outperforms our previous faster region convolutional neural network (FasterRCNN) model [54,55] in both detection accuracy and speed [22]. Images output from this model with bounding boxes are passed to a Phi-3.5-vision-instruct model to extract additional object details and contextual information, which is discussed further in the following section.

### 2.5. Vision Language Model

The Phi-3.5-vision-instrut model is a state-of-the-art multimodal system capable of processing both text and image data, making it ideal for tasks requiring a deep understanding of visual content [56]. The model is equipped with an image processor that handles up to 16 crops of the input image, allowing it to focus on different regions for more detailed analysis. Efficient resource allocation ensures optimal use of hardware, such as GPUs, to handle the large volume of images encountered in wildlife monitoring projects.

The Phi-3.5-vision-instruct model consists of 4.2 billion parameters, enabling it to efficiently manage large-scale data and complex tasks. With a context length of up to 128K tokens, the model can handle extensive sequences of text and visual data, allowing for the generation of rich, detailed descriptions from both image and textual content. While EXIF tools can extract basic metadata, such as timestamps and sensor types, the OCR capabilities of this model extend far beyond this. For example, it can read study-specific visual metadata, such as configuration boards or location markers, enriching datasets with critical context often unavailable in digital metadata. These capabilities support the holistic analysis of camera trap datasets.

Trained on 500 billion tokens using 256 NVIDIA A100-80G GPUs over six days, the model’s training regime ensures high accuracy and adaptability across diverse contexts. The model’s backbone extracts features from both text and images, leveraging advanced neural network architectures to capture complex relationships within the data. Like YOLOv10, the Phi-3.5-vision-instruct model integrates large-kernel convolutions [57] and partial self-attention modules [58], enhancing its ability to focus on relevant parts of the input data [53,54]. This shared architectural optimisation across models ensures efficient processing without compromising on accuracy. Similar to YOLOv10, the Phi-3.5-vision-instruct model is deployed on a NVIDIA Triton Inference Server, ensuring efficient integration into the overall system.

**Figure 4:**
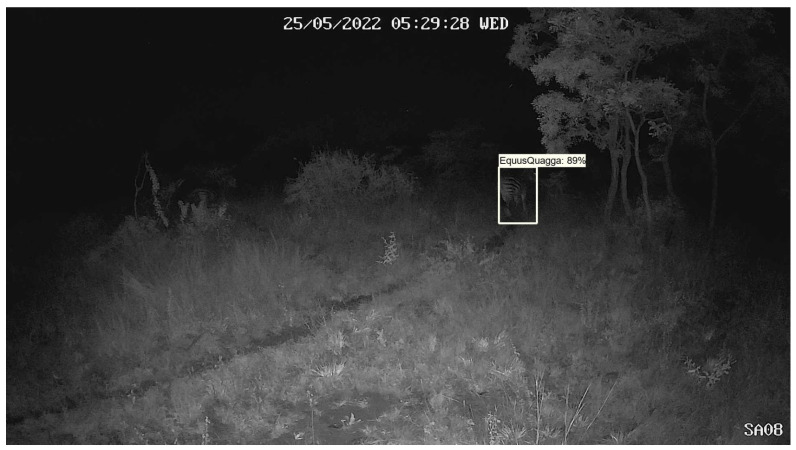
Image from Limpopo Province in South Africa showing the detection of a zebra at night using a camera trap.

The Phi-3.5 model excels in tasks requiring a deep understanding of both textual and visual data. However, when applied to camera trap images, such as those shown in Figure 4, the model struggles to reliably detect and identify specific species. To address this limitation, we rely on the fine-tuned YOLOv10 model to first detect and classify animals (fine-tuning the Phi-3.5 model to improve performance in such cases would require significant hardware resources), placing labelled bounding boxes around each detected animal. The Phi-3.5-vision-instruct model then reads these bounding box labels to identify the species, and supplements this with additional contextual information it can detect in the image, such as trees, weather, and embedded metadata text (e.g., 25/05/2022 05:29:28 WED). This multistep process ensures the system provides comprehensive analysis, while remaining computationally feasible.

Beyond immediate contextual insights, the integration of vision–language models enables study-wide querying capabilities. By referencing and synthesising information across all processed images in a dataset, users can ask time-series questions, such as the following:

- “How many elephants were observed in January 2024, and how does this compare to January 2023?”
- “How many zebras were observed in January 2023 compared with 2024, and were they more commonly observed during the day or night?”

This functionality transforms camera trap analysis, enabling conservationists and ecologists to derive meaningful insights without the need for complex programming or database queries. By using YOLOv10-X for detection and classification and the Phi-3.5 model for context analysis, we efficiently leverage the strengths of both models to maximise efficiency and accuracy and democratise insight generation.

**Figure 5:**
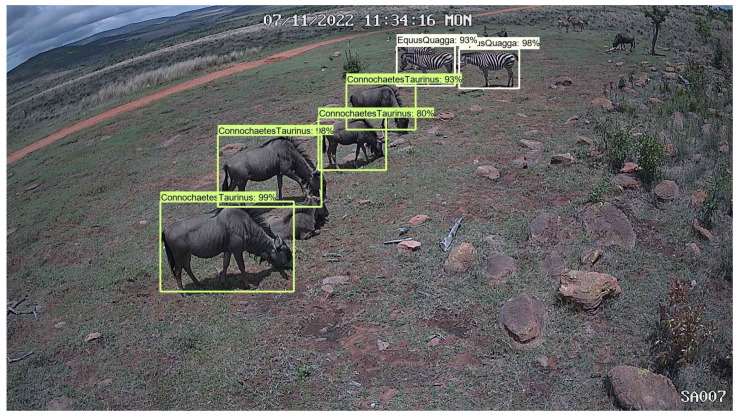
Image from Limpopo Province in South Africa showing the detection of a multiple blue wildebeest and zebras using a camera trap.

Using Figure 4 as an example, the Phi-3.5-vision-instruct model identifies the species as *Equus quagga* (zebra), the camera ID as SA08, and the time and date as 25 May 2022, 05:29:28 WED. It also infers that the image was taken in the dark in a wooded environment based on the presence of trees and grass. This contextual information, combined with object detection results, provides a more complete understanding of the scene. In Figure 5, the model detects four *Connochaetes taurinus* (blue wildebeest) and two *Equus quagga* (plains zebra), offering useful data for species abundance estimation or population dynamics.

### 2.6. Retrieval-Augmented Generation (RAG)

For information not directly available from the image—such as species weight, IUCN status, or other biological facts—the system integrates RAG to retrieve knowledge from external sources. This ensures the system delivers not only image-based insights but also enriched external data, enhancing its overall utility for conservationists. In this study, the RAG component is implemented using LangChain [59] and sources external information from Wikipedia based on image-extracted data. For example, when queried with “What animal is in this image, how much does it weigh, and what is its IUCN status?”, the system generates relevant keywords from the Phi-3.5-vision-instruct model, which are then used to search for Wikipedia documents. The retriever gathers up to 20 Wikipedia pages, which are split into smaller passages for precise extraction. Facebook AI Similarity Search (FAISS) ranks these passages based on relevance [45]. By controlling the number of retrieved passages and the chunk size, the system ensures that the most relevant information is selected. Combining this external knowledge with image analysis provides the basis for vision question answering.

### 2.7. Visual Question Answering

During the visual question answering stage, the system generates a comprehensive answer to questions by integrating visual analysis with the retrieved external knowledge. The information from the retrieved passages is synthesised with the visual analysis, resulting in detailed, contextually enriched responses. The system returns a tuple (an array containing the final answer, the keywords used for retrieval, and the selected passages), ensuring transparency in how the answer was formulated. This transparency is crucial for researchers, developing trust in the system’s outputs.

### 2.8. Automatic Reporting

The final stage in the methodology includes the integration of an automated reporting system. Once the images are processed by the object detection and vision–language models, and a detailed context is generated, the complete information is submitted to a Llama-2-7b-hf model along with user queries, which outputs Alpaca formatted responses for use in automatic reporting [60]. Using natural language inputs, users can extract tailored insights, such as time-series analysis or cross-section comparisons across species, habitats, or time periods and incorporate these as part of the report writing phase.

For example, stakeholders can ask the following:

- “How has the population of giraffes fluctuated between 2023 and 2024?”
- “What species were observed at night in the dry season verses the wet season?”

This dual functionality—combining automated reporting with interactive querying—simplifies access to actionable insights and supports informed decision making in conservation efforts.

### 2.9. Evaluation Metrics

To assess the performance of the YOLOv10-X object detection model and the Phi-3.5-vision-instruct model, we employed several key metrics, including precision, recall, F1-score, mAP, IoU, and BERTScores for evaluating generated answers against ground truth answers. Precision measures the proportion of true positives (correctly identified animals) among all positive predictions, reflecting the model’s classification accuracy. Recall evaluates the model’s ability to identify all relevant animals within images, calculating the proportion of true positives out of the total actual number of animals present. The F1-score balances precision and recall, providing a more comprehensive evaluation, particularly in scenarios where minimising false positives is crucial.

mAP is a key metric in object detection, measuring the average precision across all classes at various IoU thresholds—IoU quantifies the overlap between the predicted and actual bounding boxes, with a high IoU value indicating more accurate localisation. mAP provides a comprehensive evaluation of the model’s ability to detect and label animals accurately.

For evaluating the system’s answering capabilities, we used BERTScore, which measures precision, recall, and F1 to assess the quality of generated answers and ground truth answers [61]. This metric determines how closely the generated answers align with expected responses, particularly when visual data are enriched with external knowledge.

Each of these metrics contributes to a detailed understanding of the models’ strengths and weaknesses, ensuring a thorough evaluation across species detection, classification, and contextual information retrieval tasks.

## 3. Results

This section presents the results, which are structured around the system’s multistage approach, integrating object detection, vision language modelling, and RAG to deliver detailed, contextually rich descriptions of wildlife. Each component is evaluated based on its accuracy, robustness, and contribution to the overall system’s effectiveness.

### 3.1. Training Results for the Sub-Saharan Model

**Figure 6:**
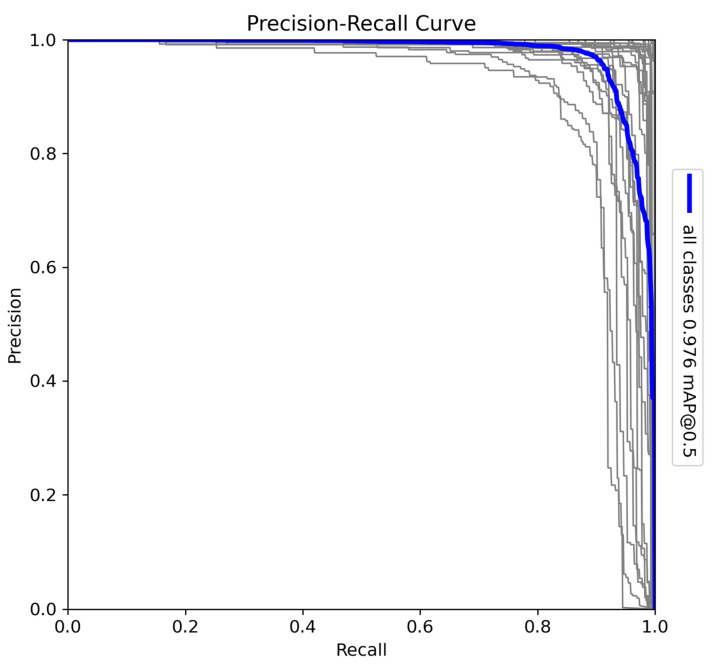
Precision–recall (PR) curve for the YOLOv10-X model trained on 29 Sub-Saharan African species, vehicles, and human subjects.

The YOLOv10-X architecture was trained to detect and classify 29 species in Sub-Saharan Africa, along with vehicles and human subjects. The dataset includes a diverse range of fauna, such as *Acinonyx jubatus* (cheetah), *Panthera leo* (lion), and *Loxodonta africana* (African elephant), presenting challenges due to the variation in morphology, size, and behaviour among species. The precision–recall (PR) curve (Figure 6) shows a mAP of 0.97 at a 0.5 IoU threshold, reflecting high detection accuracy across all classes. Precision–recall curves are essential for evaluating object detection tasks, as they illustrate the trade-off between detecting all relevant objects (recall) and avoiding false positives (precision). Additionally, F1–confidence curves and confusion matrices provide valuable insight into the model’s performance across various confidence thresholds and help identify misclassifications across species.

**Figure 7:**
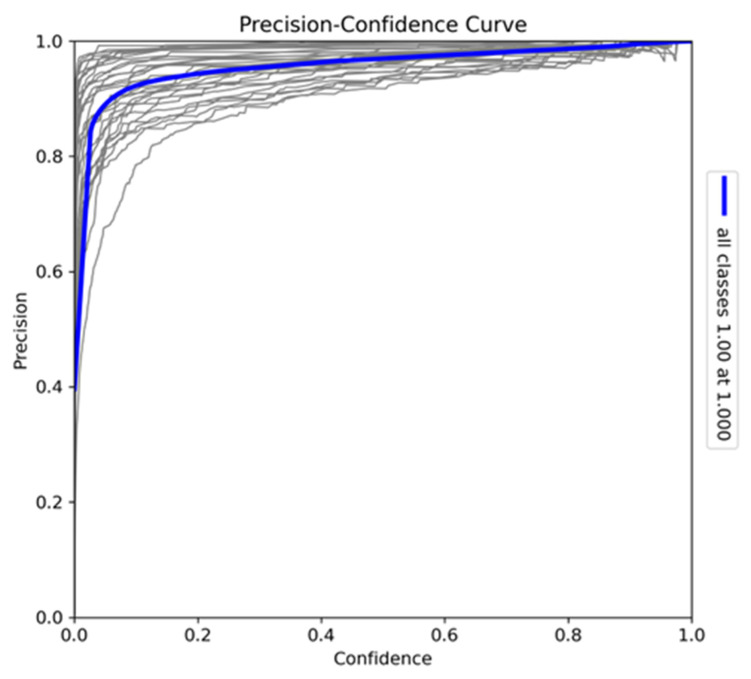
Precision–confidence curve for the model trained on Sub-Saharan African species, vehicles, and human subjects.

The precision–confidence curve (Figure 7) provides insights into the reliability of the YOLOv10-X model’s predictions across all classes. The curve demonstrates that the model achieves high precision even at low confidence thresholds, with predictions remaining accurate at moderate confidence levels. At the maximum confidence level of 1.0, the model achieves perfect precision. Variations in individual class curves, particularly for more visually similar species, indicate classification challenges, but the strong correlation between precision and confidence confirms the model’s reliability in making accurate detections across different confidence levels.

**Figure 8:**
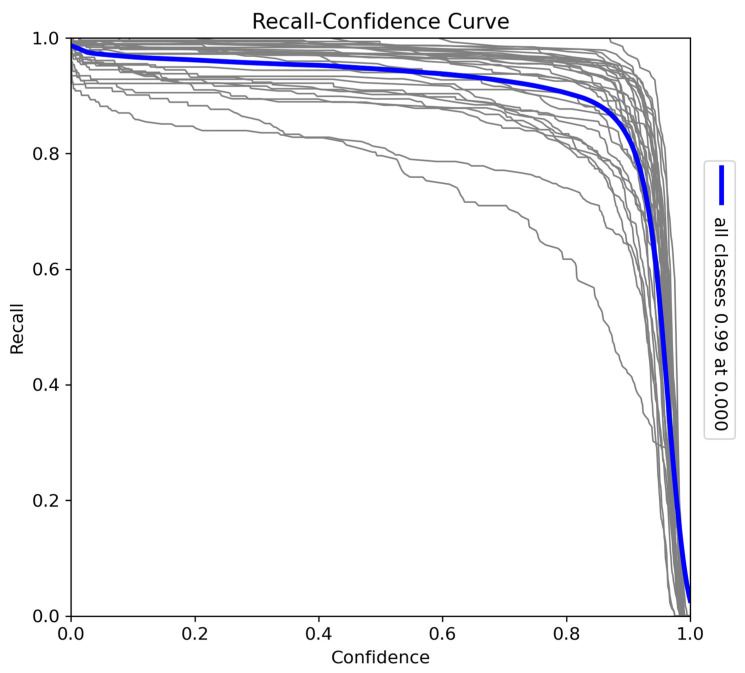
Recall–confidence curve for the model trained on Sub-Saharan African species, vehicles, and human subjects.

The recall–confidence curve (Figure 8) illustrates the trade-off between recall and confidence thresholds. At lower confidence thresholds, the model achieves near-perfect recall (0.99), indicating that it captures almost all true positives when not constrained by confidence. However, as the confidence threshold increases, recall declines, with a sharp drop near the highest confidence levels, where precision is prioritised. Variations in individual species curves suggest that certain species may benefit from more relaxed confidence thresholds to improve recall (particularly species such as *Bunolagus monticularis,* which are more difficult to detect than larger animals). Despite this trade-off, the model demonstrates strong overall recall performance, ensuring comprehensive detection coverage at lower confidence thresholds.

**Figure 9:**
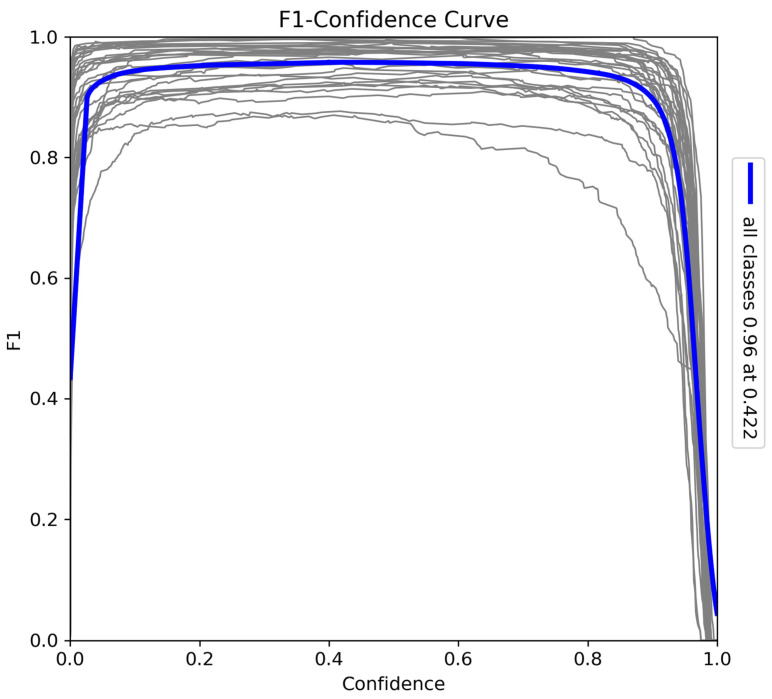
F1–confidence curve for the model trained on Sub-Saharan African species, vehicles, and human subjects.

The F1–confidence curve (Figure 9) provides a comprehensive evaluation of the model’s balance between precision and recall across various confidence thresholds. The F1-score peaks at 0.96 when the confidence threshold is 0.42, indicating optimal performance at this level. While the score remains high over a broad range of confidence levels, a sharp decline occurs near the maximum confidence threshold due to missed instances as recall decreases. Nevertheless, the model consistently achieves a strong F1-score, highlighting its effectiveness in balancing precision and recall for accurate detection tasks.

**Figure 10:**
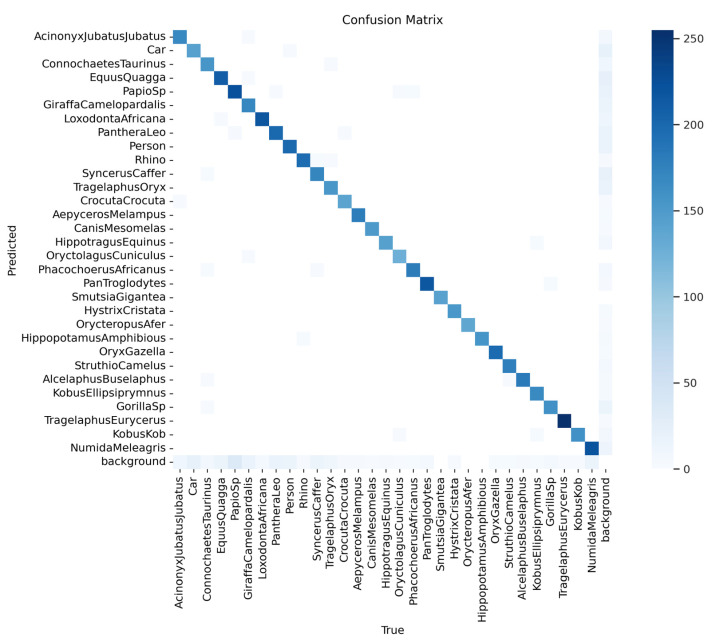
The confusion matrix provides a detailed analysis of the model’s classification performance across all Sub-Saharan African species, vehicles, and human subjects.

The confusion matrix (Figure 10) provides a detailed analysis of the model’s classification performance across the Sub-Saharan African species dataset. A strong diagonal indicates correct predictions, with high values showing the accurate identification of most species. The highest frequencies are observed for commonly occurring classes, like *Acinonyx jubatus* and *Loxodonta africana*, reflecting the model’s effectiveness with these species. Off-diagonal cells represent minimal misclassifications, likely due to visual similarities between species. Overall, the matrix confirms the model’s strong classification accuracy across the majority of classes, with relatively low rates of misclassification.

### 3.2. Results for Vision–Language Model Without YOLOv10-X Object Detection Support

This section presents the performance results for the Phi-3.5-vision-instruct model using the 602 independent camera trap images without object detection support. In this scenario, the model relies solely on its vision-based capabilities to detect and classify animals. As shown in Table 1, the model demonstrates high precision across most classes, with some achieving perfect precision (1.00). However, the recall values are significantly lower for several species, indicating difficulties in identifying all instances of the species. Despite overall accuracy exceeding 90% in most cases, the recall and F1-score metrics highlight areas where the model struggles with complete identification, reflecting the limitations of operating without prior localisation and object labelling.

Class-wise, the model’s performance varies significantly. For example, the *Canis mesomelas* (black-backed jackal) achieves an accuracy of 98.33%, but its low recall (0.20) results in an F1-score of 0.33, highlighting difficulties in consistently detecting this species. In contrast, the *Syncerus caffer* (African buffalo) (recall 0.63) and *Struthio camelus *(common ostrich) (recall 0.60) show stronger performance, with F1-scores of 0.77 and 0.75, respectively. Similarly, the *Gorilla* sp. performs well, with a recall of 0.68 and an F1-score of 0.81, demonstrating the model’s effectiveness in identifying more visually distinct species. It is important to note that some animals, such as gorillas, are grouped at the genus level (*Gorilla* sp.) rather than by subspecies. This is because object detection models often struggle to differentiate between closely related subspecies due to their high visual similarity. For example, distinguishing between the *Gorilla beringei* (eastern gorilla) and the *Gorilla gorilla* (western gorilla) would require finer-grained visual features than those typically captured in camera trap data. As a result, grouping by genus ensures more reliable detection and avoids introducing additional errors into the analysis.

The model struggles with certain species, particularly *Rhinocerotidae*, *Papio* sp., and *Tragelaphus oryx* (common eland). The *Rhinocerotidae* (rhinoceros) suffers from very low precision (0.08) despite a higher recall (0.66), leading to an F1-score of 0.15. The *Papio* sp. similarly displays a low recall of 0.07, resulting in an F1-score of 0.13. For the *Tragelaphus oryx*, the model achieves a lower precision of 0.83 and recall of 0.23, reflecting its inconsistent ability to detect these species accurately. These challenges highlight the model’s difficulty with less distinguishable species (caused by night, occlusion, or distance) or those underrepresented in the dataset.

**Figure 11:**
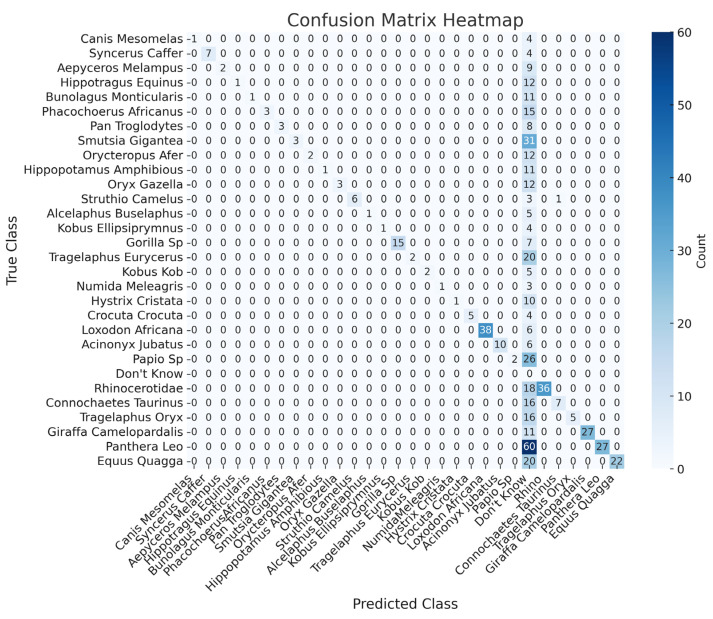
The confusion matrix provides a detailed breakdown of the classifications made by the Phi-3.5-vision model when applied to raw images without YOLOv10-X object detection support.

Overall, the model achieves high precision and accuracy. However, it struggles with consistent identification in more challenging cases, as evidenced by lower recall and F1-scores for certain species. The confusion matrix in Figure 11 provides a detailed breakdown of the classifications made by the Phi model. As indicated by the confusion matrix and supported by the results in Table 1, the model performed well for certain species. However, other species show more frequent misclassifications. This analysis provides valuable insights into the strengths and weaknesses of the Phi-3.5 across different species in challenging camera trap images. These limitations, particularly for visually similar animals, underscore the necessity of incorporating object detection for improved accuracy.

### 3.3. Results for Vision–Language Model with OD Support

This section presents the performance of the Phi-3.5-vision-instruct model with object detection support for animal identification. The labelled images, from YOLO, are processed by the Phi-3.5 model, which uses its optical character recognition (OCR) capabilities to identify the animals based on the bounding box text. This two-step method overcomes the model’s earlier limitations.

Compared to the previous set of results, object detection support significantly reduces misclassifications and increases overall accuracy (Table 1), particularly in cases where the model previously struggled with lower recall and species identification challenges. For example, species like the *Tragelaphus eurycerus *(bongo) and *Papio* sp. (baboons), which exhibited lower recall and F1-scores, now show improved identification with more balanced precision and recall pairs. Species such as the *Hippopotamus amphibius *(common hippopotamus), *Oryx gazella *(South African oryx), *Alcelaphus buselaphus *(hartebeest), *Gorilla* sp. (gorilla), *Kobus kob *(African antelope), and *Numida meleagris *(helmeted guineafowl) achieve perfect scores (1.00) for accuracy, precision, recall, and F1-score, showcasing the model’s precise identification capabilities without misclassification. Other species, including the *Syncerus caffer *(African buffalo) and *Struthio camelus *(common ostrich), also perform well, with recall values of 0.84 and 0.80, respectively, and F1-scores of 0.91 and 0.88, reflecting a strong balance between precision and recall. However, certain species, such as the *Tragelaphus eurycerus *(bongo) and *Papio* sp. (baboon), present challenges, even with object detection support (probably due to animals caught in the camera trap, at distance, at night). For instance, *Tragelaphus eurycerus *(bongo) shows a recall of 0.09 and an F1-score of 0.16, reflecting ongoing difficulties in reliably identifying this species. Similarly, *Papio* sp. (Baboon) achieves a recall of 0.44 and an F1-score of 0.61, indicating challenges in classification for that class. Additionally, for the *Rhinocerotidae *(rhinoceros), the model shows a high recall of 0.98 but struggles with precision (0.46), resulting in a modest F1-score of 0.62. This suggests that, while the model captures a large number of true positives for the *Rhinocerotidae *(rhinoceros), it remains prone to misclassifications (likely caused by images captured at night, which are more difficult to classify).

Overall, the model with object detection support exhibits excellent performance across most species, particularly those with distinct morphometric characteristics, with many species achieving near-perfect metrics as shown in Table 1. The integration of object detection has significantly reduced misclassification rates and improved the model’s ability to accurately identify animals in challenging camera trap images.

**Figure 12:**
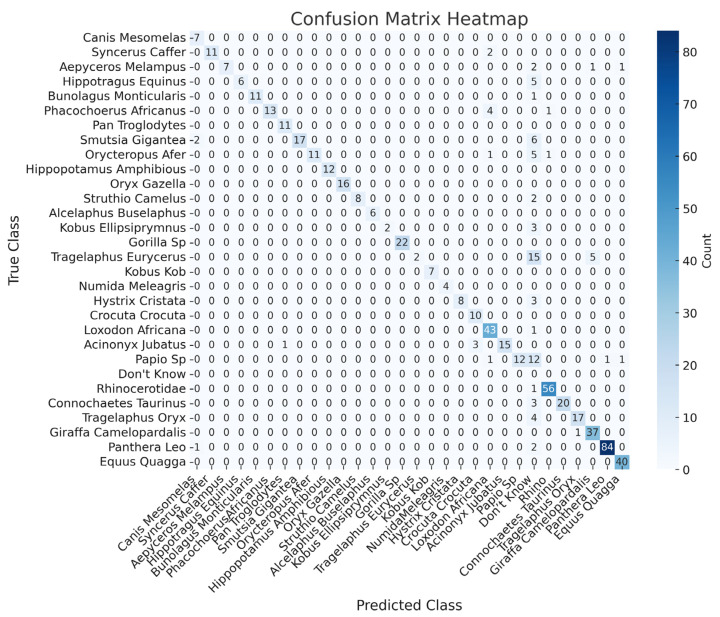
Confusion matrix for the Phi-3.5 model using the bounding boxes from the test case images.

The confusion matrix (Figure 12) offers a comprehensive breakdown of the Phi-3.5 model’s performance with OD support. The matrix highlights that the model performs exceptionally well for several species, with most true instances aligning along the diagonal—indicative of correct classification. For example, the *Canis mesomelas *(black-backed jackal) has 7 true positives, and the *Syncerus caffer *(African buffalo) has 11 true positives, showing minimal confusion with other species.

Despite these successes, some misclassifications are observed, particularly with species such as the *Papio* sp. (baboon), *Phacochoerus africanus *(common warthog), and *Smutsia gigantea *(giant ground pangolin). For instance, the *Papio* sp. (baboon) is occasionally misclassified as the *Acinonyx jubatus* (cheetah) or given a “don’t know” response. Similarly, the *Phacochoerus africanus* (common warthog) is sometimes incorrectly classified as the *Syncerus caffer *(African buffalo). Additionally, the *Smutsia gigantea *(giant ground pangolin) exhibits slight confusion with the *Canis mesomelas *(black-backed jackal) in certain cases.

Overall, the confusion matrix illustrates that, while the model achieves high accuracy for many species, certain species remain challenging to classify. The integration of object detection has reduced some of these errors, but there is still room for improvement, particularly in handling species that are more difficult to distinguish.

### 3.4. Results for Retrieval-Augmented Generation

In this section, we evaluate the capabilities of the Phi-3.5 model within the RAG framework. Building on its success in species identification with object detection support, we now assess how the model synthesises detailed information about the identified species using external knowledge and compare its outputs with ground truth responses (provided by conservationists).

The results presented (see Table 2 and Appendix A) show the BERTScores (precision, recall, and F1-score) and the images used to evaluate the similarity between the model-generated answers and the ground truth. These scores assess the contextual relevance and accuracy of the model’s answers, as compared to the ground truth. For instance, the question “Was the image taken during the day or night?” achieved high precision (0.94), recall (0.91), and F1-score (0.91), indicating that the model was highly effective in interpreting time and environmental factors. Similarly, for questions related to species identification and IUCN conservation status, the model produced strong results, with an F1-score of 0.93, underscoring its ability to accurately retrieve and present relevant species-specific information from external sources.

More complex reasoning or comparison-based questions, such as “How does the species identified in the image compare to other species in the same habitat?” (F1-score: 0.86) and “What are the known predators or threats to the species?” (F1-score: 0.85), also demonstrated strong performance. By focusing on the comparison between the generated answers and ground truth, the evaluation highlights the system’s ability to deliver accurate and reliable information across a wide range of ecological and environmental questions. The variation in scores—particularly between fact-based questions and those requiring broader ecosystem-level insights (e.g., “What is the species’ role in the ecosystem?” F1-score: 0.85)—shows that, while the system performs well, additional refinement may be needed for more complex, multifaceted inquiries.

Overall, the results indicate that the Phi3.5 model with RAG is highly proficient in generating accurate and informative answers when compared to the ground truth. By effectively leveraging information retrieved from bounding box labels and supplementary sources, the system shows significant potential in enhancing wildlife monitoring and conservation efforts.

### 3.5. Automated Reporting

**Figure 13:**
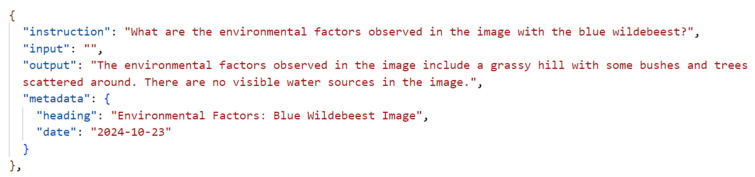
Alpaca JSON format showing the question–answer pairs.

To streamline the analysis of camera trap data, an automated reporting system was developed. This system integrates the outputs of deep learning models to efficiently generate structured reports. After images are processed by the Phi-3.5 model and a user generates a question, the Llama-2-7b-hf model is employed to automatically generate question answer pairs using the Phi-3.5 derived information. This process leverages the Alpaca format for question–answer pair generation, ensuring consistency and clarity in the presentation of the extracted data (see Figure 13).

**Figure 14:**
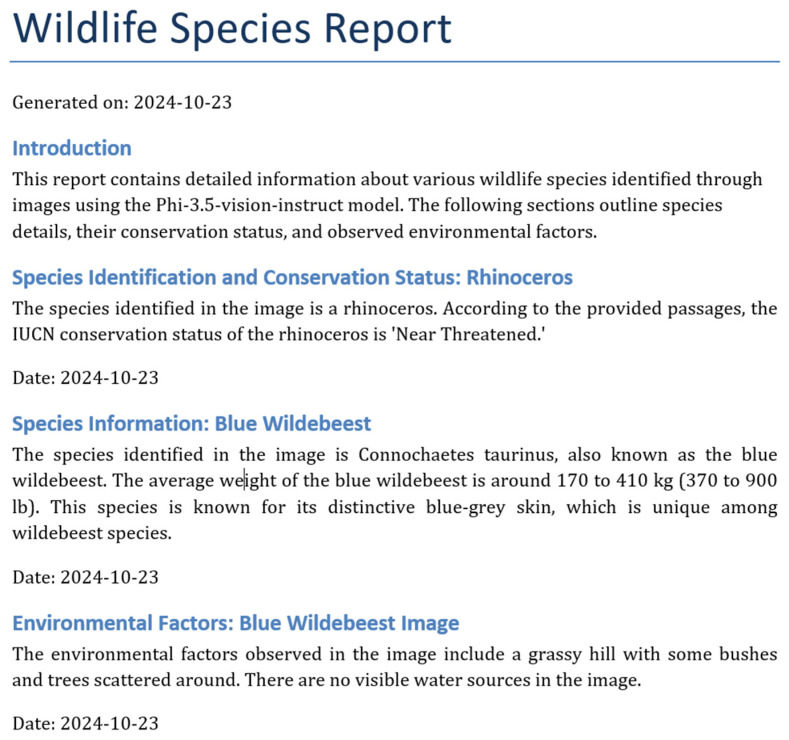
Sample report using Alpaca Q&A.

This function is applied to the entire dataset of camera trap images, resulting in a comprehensive collection of species-specific analyses. In order to further enhance the utility of these outputs, the Alpaca-formatted data are converted into a structured report using the Python docx library (see Figure 14). This conversion process translates the JSON data [62] into a well-organised Word document that is accessible to interested stakeholders. The report includes information on species identification, conservation status, environmental factors, and behavioural predictions, from all camera traps, providing a holistic view of each observation. Note that this is a simplistic example that demonstrates the applicability of the approach and not a useful report suitable for stakeholder tasks.

## 4. Discussion

The results from this study highlight both the strengths and weaknesses of the Phi-3.5 model, particularly in challenging scenarios involving low-quality camera trap images. In the initial set of experiments, where the model processed images without object detection support, it became evident that identifying species in such conditions is inherently difficult (likely as the model was not initially trained on camera trap images). While the model demonstrated high precision for certain species—such as *Syncerus caffer* and *Struthio camelus*, which achieved perfect precision (1.00; Table 1)—it struggled significantly with recall. For instance, *Canis mesomelas* had a recall of just 0.20, leading to a low F1-score of 0.33, underscoring the model’s difficulty in consistently identifying species without localisation assistance.

With the integration of object detection, the performance of the Phi-3.5 model improved significantly across all key metrics. By using the YOLOv10-X model to localise and classify animals first within images and combining the results with the optical character recognition capabilities of the Phi-3.5 model for species identification, the system achieved substantial gains. For example, the F1-score for *Canis mesomelas* rose sharply from 0.3333 to 0.82 (Table 1), once including our multimodal approach. Similarly, *Syncerus caffer*, which already performed well, saw its F1-score improve to 0.91. Notably, some species, such as *Hippopotamus amphibius* and *Oryx gazella*, achieved perfect scores (1.00) across accuracy, precision, recall, and F1-score, underscoring the effectiveness of combining object detection with vision–language models.

By focusing on interpreting labels within bounding boxes, the Phi-3.5 model bypassed many of the challenges associated with direct image analysis, significantly reducing the rate of misclassification and improving accuracy. Additionally, the model could extract peripheral information, such as environmental features like trees and water sources, as well as metadata, such as time stamps and camera IDs. This capability extends beyond traditional metadata tools, like EXIF, which can only retrieve digital metadata, by enriching datasets with study specific contextual details often embedded in the images themselves.

Furthermore, the integration of vision–language models transforms camera trap datasets into a rich source for temporal and spatial analysis. By leveraging study-wide querying capabilities, users can track trends across years, seasons, or habitats without requiring programming expertise. For example, stakeholders can ask “How has the population of the bongo changed from 2022 to 2024?” or “What proportion of lions were observed during the day versus night over the past year?” These advanced querying functionalities, combined with automated reporting, provide a comprehensive framework for conservation analysis that is both intuitive and powerful.

Another less obvious benefit of incorporating the YOLO model is its low inference cost and its ability to remove blank images. Since blank images make up approximately 80% of camera trap datasets (based on our own empirical studies), removing these using the YOLOv10-X model allows for a more efficient and cost-effective solution, reducing computational overheads and enabling the faster processing of meaningful data.

However, one major issue encountered during the study was the inconsistent presentation of bounding boxes and text labels. Nonstandard colour combinations, such as white text on pink backgrounds, made it difficult for the OCR component to accurately read the labels, leading to species misidentifications. Additionally, certain images—especially higher resolution ones—featured thinner bounding boxes and smaller text, which further complicated label readability. In these instances, the Phi-3.5 model struggled with text recognition, resulting in misclassifications and missed identifications. For example, even with object detection, the *Rhinocerotidae* continued to present challenges, achieving a precision of 0.46 (Table 1) and an F1-score of 0.62, likely due to these text readability issues and poor visibility in images.

Despite the improvements from object detection, certain species—such as *Papio* sp. and *Tragelaphus eurycerus*—continued to exhibit relatively low performance (the later images were of a much higher resolution). While *Papio* sp. showed some improvement, it only achieved a recall of 0.44 and an F1-score of 0.61, reflecting ongoing challenges in accurate species identification. Similarly, *Tragelaphus eurycerus* had a recall of 0.0909 and an F1-score of 0.16, underscoring the model’s persistent difficulty in reliably classifying these species, particularly those with fewer samples or more ambiguous visual characteristics.

Building on the successful integration of the two models, the use of RAG further demonstrated the system’s capacity to further enrich species identification by incorporating external contextual information. By sourcing data from Wikipedia, the model provided supplementary insights such as average weight, conservation status, and environmental context. This extended the model’s functionality beyond simple species identification, adding significant ecological and biological depth to the analysis. Using the Q&A feature over this integration, F1-scores ranged between 0.82 and 0.94 for various answer–ground-truth comparisons; for example, its precision for identifying species and their IUCN conservation status reached 0.95, with a recall of 0.91 and an F1-score of 0.9382. Similarly, its handling of environmental factors, like determining whether an image was taken during the day or night, yielded an F1-score of 0.91. These scores indicate that the RAG-enhanced model can accurately combine object detection with species identification and contextual knowledge, enriching the system’s overall output.

From a conservation perspective, this system demonstrates significant potential to streamline workflows and improve data accessibility for stakeholders. The ability to integrate species identification with contextual information, such as conservation status and environmental context, could inform better decision making for habitat protection and species management. For instance, the identification of invasive species or degraded habitats from camera trap images could help conservationists allocate resources more efficiently. However, further work is needed to align these outputs with conservation priorities, such as automated biodiversity metrics, population density estimations, and species movement tracking. Building on this proof of concept, the system could be enhanced with tailored datasets and integration into larger conservation workflows, ultimately supporting real-time monitoring and preventative conservation actions.

## 5. Conclusions

This study enhances camera trap image analysis by integrating advanced AI models. The YOLOv10-X object detection model enables precise animal detection and localisation, while the Phi-3.5-vision-instruct model incorporates vision–language capabilities for species identification and the extraction of peripheral environmental data. Additionally, the integration of RAG further enriches the system by retrieving detailed species-specific information, such as IUCN status, average weight, and environmental context, from external knowledge sources, like Wikipedia.

Beyond these functionalities, the hybrid system transforms how camera trap datasets are analysed by facilitating intuitive queries and time-series analysis. By synthesising information across entire studies, the system allows stakeholders to ask questions, such as “How does species distribution vary by season?” or “What trends are observable in population dynamics across years?” This capability eliminates the need for complex coding or dataset queries, democratising access to insights for conservationists and ecologists.

Moreover, the OCR capabilities of the system extend traditional metadata extraction by reading visual information, such as configuration boards and study specific notes embedded in the images. This feature complements digital metadata tools, like EXIF, providing a richer, more holistic understanding of the dataset.

These advancements pave the way for a new era of camera trap analysis, where powerful, AI-driven insights are accessible to a broad range of users. While this is a position paper outlining the potential of this system, future work will focus on validating these applications in real-world conservation studies, refining the system to further support proactive biodiversity management.

This combined approach demonstrates significant improvements in species classification, particularly in challenging low-quality images where traditional models often fail. The high accuracy, precision, recall, and F1-scores across most species validate the effectiveness of the methodology. Moreover, the RAG component adds additional contextual richness by providing supplementary insights, which are critical for informed wildlife management decisions. The system’s potential integration with emerging technologies, such as drone-based monitoring or satellite imagery, also paves the way for broader conservation applications, enhancing the utility of this framework.

The inclusion of an automated reporting system, while rudimentary in its current implementation, demonstrates the potential for automatically generating structured reports based on model outputs. By providing stakeholders with immediate access to information, this system could significantly reduce manual effort. By continuing to refine the system, this integrated AI approach offers a scalable, efficient, and cost-effective solution to wildlife conservation. It provides deeper insights, enabling more timely and effective conservation efforts on a global scale. Ultimately, the combination of object detection, vision–language models, and RAG offers a transformative advancement in wildlife monitoring and species management.

However, despite the encouraging results, several challenges remain. The system exhibits limitations in visually complex environments and with species that exhibit high visual similarity. For example, distinguishing between subspecies of antelope, such as the *Aepycerous melampus *(impala) and *Kobus ellipsiprymnus *(waterbuck), is particularly challenging due to their morphological similarities, especially in low-resolution camera trap images. Similarly, environmental factors, such as poor lighting, occlusions, and cluttered backgrounds, can reduce detection and classification accuracy.

Another limitation stems from the reliance on bounding box labels for species identification. The accuracy of the Phi-3.5 model’s OCR capabilities is contingent upon the clarity and quality of these labels, and issues such as small text or nonstandard colours can lead to misclassifications. Future work will explore alternative approaches to mitigate these issues, such as enhancing image preprocessing techniques or leveraging higher-resolution datasets.

While the hybrid system demonstrates robust performance in many scenarios, addressing these limitations is critical for expanding its applicability in challenging conservation settings. By refining the model architecture and exploring supplementary data sources, such as audio or thermal imaging, the system could further improve species differentiation and environmental adaptability.

Another promising future direction involves a more streamlined approach where, instead of passing the image with bounding boxes and labels to the Phi-3.5 model for species identification, the system could extract this information from a structured database (e.g., SQL). By directly informing the Phi-3.5 model of the presence and identity of the species, the model could then focus solely on providing additional contextual information using its internal vision capabilities. This approach could bypass the limitations of text readability and bounding box inconsistencies. Implementing such a solution would require advanced prompt engineering, an area not covered extensively in this study, which employed only basic prompt techniques. Further investigation into optimised prompt engineering could greatly improve the system’s overall performance and facilitate more accurate contextual analysis.

## Figures and Tables

**Table 1:** Accuracy, precision, recall, and F1-score results for all Sub-Saharan species following Phi3.5-vision model processing without and with object detection support.

		Without OD				With OD			
Class	Common Name	Acc	Pre	Rec	F1	Acc	Pre	Rec	F1
*Canis mesomelas*	Black-backed jackal	0.98	1.00	0.20	0.33	0.99	0.70	1.00	0.82
*Syncerus caffer*	African buffalo	0.98	1.00	0.63	0.77	0.99	1.00	0.84	0.91
*Aepyceros melampus*	Impala	0.96	1.00	0.18	0.30	0.99	1.00	0.63	0.77
*Hippotragus equinus*	Roan antelope	0.95	1.00	0.08	0.14	0.99	1.00	0.54	0.70
*Bunolagus monticularis*	Bushman rabbit	0.95	1.00	0.08	0.15	0.99	1.00	0.91	0.95
*Phacochoerus africanus*	Common warthog	0.94	1.00	0.16	0.28	0.99	1.00	0.72	0.83
*Pan troglodytes*	Chimpanzee	0.96	1.00	0.27	0.42	0.99	0.91	1.00	0.95
*Smutsia gigantea*	Giant ground pangolin	0.88	1.00	0.08	0.16	0.98	1.00	0.68	0.80
*Orycteropus afer*	Aardvark	0.95	1.00	0.14	0.25	0.98	1.00	0.61	0.75
*Hippopotamus amphibious*	Common hippopotamus	0.95	1.00	0.83	0.15	1.00	1.00	1.00	1.00
*Oryx gazella*	South African oryx	0.95	1.00	0.20	0.33	1.00	1.00	1.00	1.00
*Struthio camelus*	Common ostrich	0.98	1.00	0.60	0.75	0.99	1.00	0.80	0.88
*Alcelaphus buselaphus*	Hartebeest	0.97	1.00	0.16	0.28	1.00	1.00	1.00	1.00
*Kobus ellipsiprymnus*	Waterbuck	0.98	1.00	0.20	0.33	0.99	1.00	0.40	0.57
*Gorilla* sp.	Gorilla	0.97	1.00	0.68	0.81	1.00	1.00	1.00	1.00
*Tragelaphus eurycerus*	Bongo	0.92	1.00	0.09	0.16	0.96	1.00	0.09	0.16
*Kobus kob*	African antelope	0.97	1.00	0.28	0.44	1.00	1.00	1.00	1.00
*Numida meleagris*	Helmeted guineafowl	0.98	1.00	0.25	0.40	1.00	1.00	1.00	1.00
*Hystrix cristata*	Crested porcupine	0.95	1.00	0.09	0.16	0.99	1.00	0.72	0.84
*Crocuta crocuta*	Spotted hyena	0.98	1.00	0.55	0.71	0.99	0.76	1.00	0.86
*Loxodonta africana*	African elephant	0.97	1.00	0.86	0.92	0.98	0.84	0.97	0.90
*Acinonyx jubatus*	Cheetah	0.97	1.00	0.62	0.76	0.99	1.00	0.78	0.88
*Papio* sp.	Baboon	0.90	1.00	0.07	0.13	0.97	1.00	0.44	0.61
*Rhinocerotidae*	Rhinoceros	0.37	0.08	0.66	0.15	0.88	0.46	0.98	0.62
*Connochaetes taurinus*	Blue wildebeest	0.93	1.00	0.30	0.46	0.99	0.90	0.86	0.88
*Tragelaphus oryx*	Common eland	0.93	0.83	0.23	0.37	0.99	1.00	0.80	0.89
*Giraffa camelopardalis*	Giraffa	0.95	1.00	0.71	0.83	0.99	0.97	0.97	0.97
*Panthera leo*	African lion	0.79	1.00	0.31	0.47	0.98	0.93	0.96	0.94
*Equus quagga*	Plains zebra	0.92	1.00	0.52	0.68	0.99	0.97	1.00	0.98

**Table 2:** Phi-3.5 and RAG Q&A results.

Question	Answer	Ground Truth	Pre	Rec	F1
**Q1. Read the label on the bounding box to identify the animal. What is the species identified in the image, and what is its IUCN conservation status?**	A1. The species identified in the image is a rhino. According to the provided passages, the IUCN conservation status of the rhinoceros is “near threatened”.	GT1. The species in the image is a rhinoceros, and its IUCN status is near threatened.	0.95	0.91	0.93
**Q2 Read the label on the bounding box to identify the animal. What is the average weight of the species identified, and does this species have any notable characteristics or behaviours?**	A2. The animal identified in the image is a *Connochaetes taurinus*, also known as the blue wildebeest. The average weight of the blue wildebeest is around 170 to 410 kg (370 to 900 lb). This species is known for its distinctive blue-grey skin, which is unique among wildebeest species. They are also known for their migratory behaviour, forming large herds that travel in search of food and water.	GT2. There are two *Connochaetes taurinus* in the image. The average weight is 250 kg (550 lb). It is highly gregarious and superbly adapted for a migratory existence.	0.87	0.85	0.86
**Q3. Was the image taken during the day or night, and what environmental factors can be observed (e.g., forest, bush, water sources)?**	A3. The image was taken during the day. The environmental factors that can be observed include a grassy hill with some bushes and trees scattered around. There are no visible water sources in the image.	GT3. The image was taken during the day. The environment is a grassland with several trees typical of an African plain.	0.94	0.91	0.91
**Q4. Read the label on the bounding box to identify the animal. How does the species identified in the image compare to other species in the same habitat in terms of size, behaviour, and diet?**	A4. The species identified in the image is the *Giraffa camelopardalis*, which is a large herbivorous mammal native to Africa. Giraffes are known for their long necks and legs, and they are the tallest mammals on Earth. They are typically found in savannas, grasslands, and open woodlands in Africa. Giraffes are also known for their unique behaviour of browsing on leaves, flowers, and fruits from tall trees, which sets them apart from other antelope species that primarily graze on grasses. In terms of diet, giraffes are herbivores and primarily feed on leaves, flowers, and fruits from trees, particularly from the Acacia tree.	GT4. The animal is a *Giraffa camelopardalis*. *Giraffa camelopardalis* are the tallest mammal with a long neck that makes it ideal for eating the leaves and twigs of acacia, mimosa, and wild apricot trees. *Giraffa camelopardalis* are herbivores.	0.87	0.86	0.86
**Q5. Read the label on the bounding box to identify animals. Can you identify other animals or objects in the image, such as nearby trees, water bodies, or structures?**	A5. In the image, there is a zebra standing in the centre of the frame. There are no other animals visible in the image. The background consists of a dirt road and some vegetation, but no water bodies or structures are visible.	GT5. The animal in the image is a Equus quagga. It is standing on a dirt track, which is surrounded by trees. There are no other animals in the image.	0.89	0.89	0.89
**Q6 Read the labels on the bounding boxes to identify animals. What animals are in the image and how many are there of each animal species identified?**	A6. The image contains three rhinoceroses.	GT6. The animals in the image are rhinoceroses. There are three of them.	0.87	0.87	0.87
**Q7. Based on the species and its habits, what predictions can be made about its activity at the time the camera trap image was taken (e.g., hunting, foraging, resting)?**	A7. Based on the species and its habits, it can be predicted that the lion is likely resting or inactive at the time the camera trap image was taken. Lions spend much of their time resting, with only intermittent bursts of activity throughout the day. The image shows a lion lying down on the grass, which supports this prediction.	GT7. The picture contains several lions—only one is fully visible. All the lions are lying down resting.	0.89	0.85	0.87
**Q8. Read the label on the bounding box around the animal to determine the species. What potential threats, either natural or human-induced, are most relevant to the species in the image, given its current IUCN status and environment?**	A8. The species in the image is a pangolin, specifically the giant pangolin (*Manis gigantea*). Given its current IUCN status as endangered, the potential threats most relevant to the species in the image include illegal, international trade in pangolin skin, scales, and meat, as well as habitat loss and fragmentation. These threats are primarily human-induced and can lead to a decline in pangolin populations.	GT8. The image contains a pangolin, which is the most hunted mammal with an IUCN status of endangered. Its main threat is humans who poach them for the meat and scales.	0.90	0.87	0.88
**Q9. Read the label on the bounding box around the animal to determine the species. What is the species role in the ecosystem, and how does its presence effect other species or the environment in the area where the image was captured?**	A9. The species in the image is the African bush elephant (*Loxodonta africana*). As a keystone species, the presence of African bush elephants in the ecosystem has a significant impact on other species and the environment. They play a crucial role in shaping the landscape by uprooting trees and undergrowth, which can transform savannah into grasslands. This creates more accessible habitat for smaller herbivores to access trees that have been mowed down by elephants. Additionally, their digging for water during dry seasons can create water holes that benefit other species in the area.	GT9. The image contains an African elephant. They maintain habitats and promote the growth of larger trees. They trample forests and dense grasslands, making room for smaller species to co-exist.	0.87	0.83	0.85
**Q10. Read the label on the bounding box around the animal to determine the species. What are the known predators or threats to the species in the image, and are there any visible indicators in the environment that suggest the presence of these threats?**	A10. The species in the image is a spotted hyena (*Crocuta crocuta*). Known predators or threats to the spotted hyena include lions (*Panthera leo*), cheetahs (*Acinonyx jubatus*), leopards (*Panthera pardus*), brown hyenas (*Parahyaena brunnea*), and African wild dogs (*Lycaon*). There are no visible indicators in the environment that suggest the presence of these threats.	GT10. The animal in the image is *Crocuta Crocuta*. Its only true predators are lions and humans. There are no visible threats in the image.	0.90	0.81	0.85

**Figure A1:**
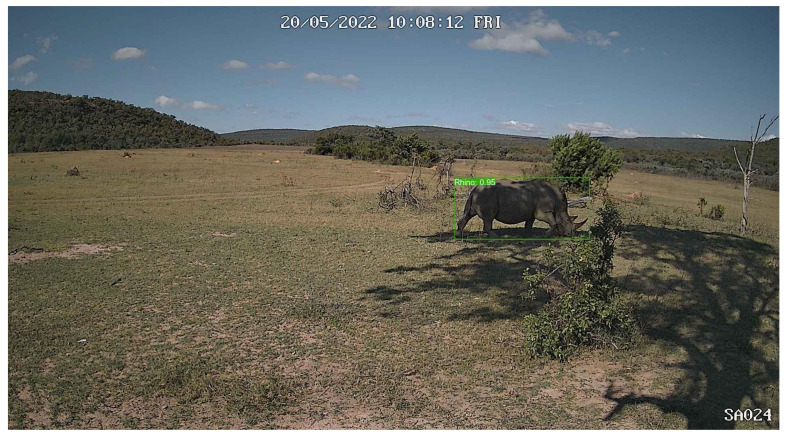
Q1. Read the label on the bounding box to identify the animal. What is the species identified in the image, and what is its IUCN conservation status?

**Figure A2:**
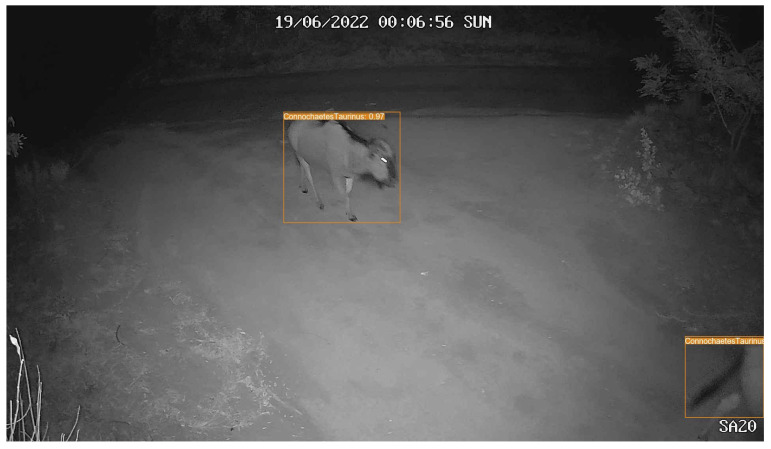
Q2 Read the label on the bounding box to identify the animal. What is the average weight of the species identified, and does this species have any notable characteristics or behaviours?

**Figure A3:**
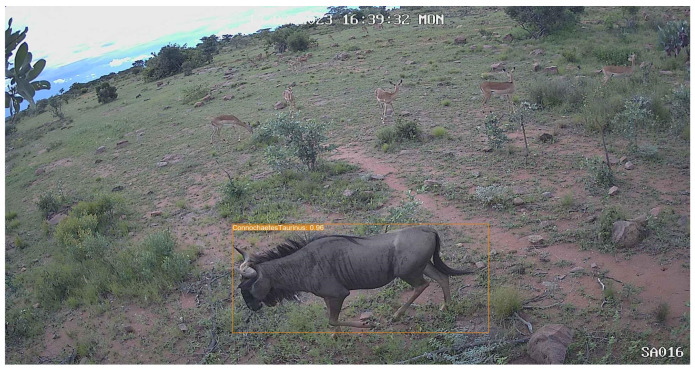
Q3. Was the image taken during the day or night, and what environmental factors can be observed (e.g., forest, bush, water sources)?

**Figure A4:**
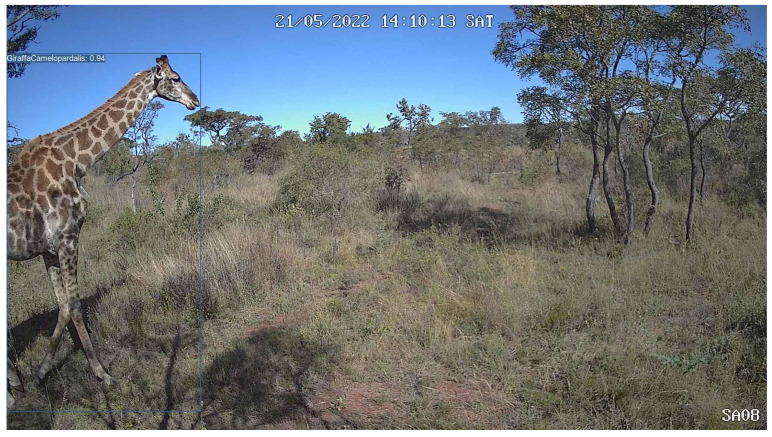
Q4. Read the label on the bounding box to identify the animal. How does the species identified in the image compare to other species in the same habitat in terms of size, behaviour, and diet?

**Figure A5:**
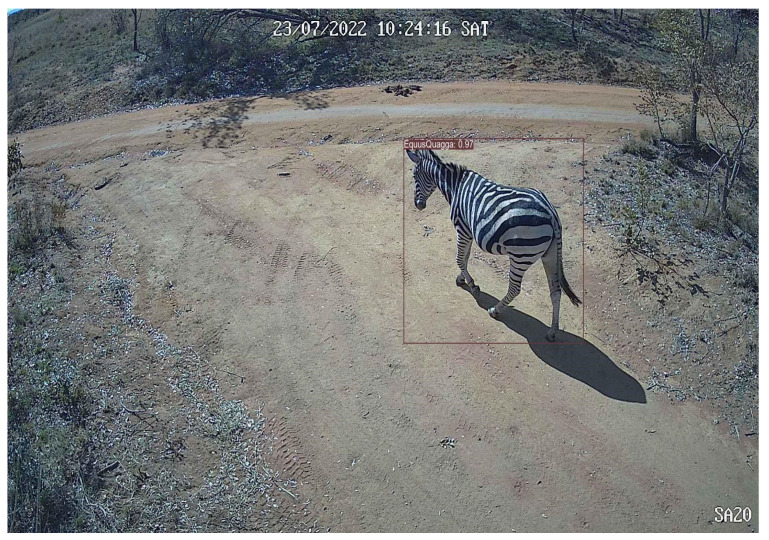
Q5. Read the label on the bounding box to identify animals. Can you identify other animals or objects in the image, such as nearby trees, water bodies, or structures?

**Figure A6:**
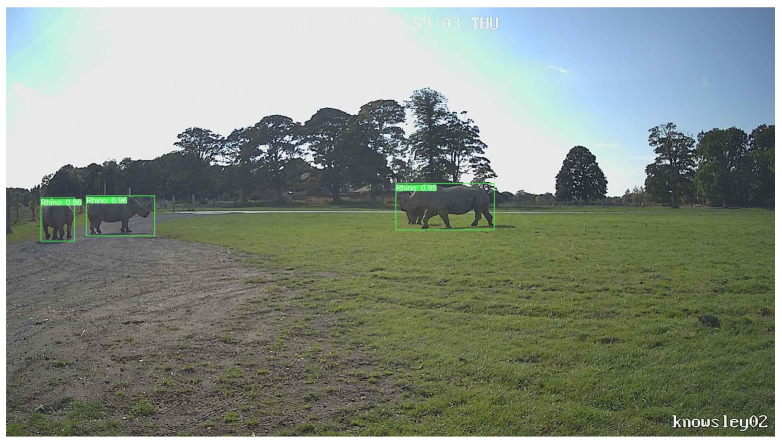
Q6. Read the labels on the bounding boxes to identify animals. What animals are in the image and how many are there of each animal species identified?

**Figure A7:**
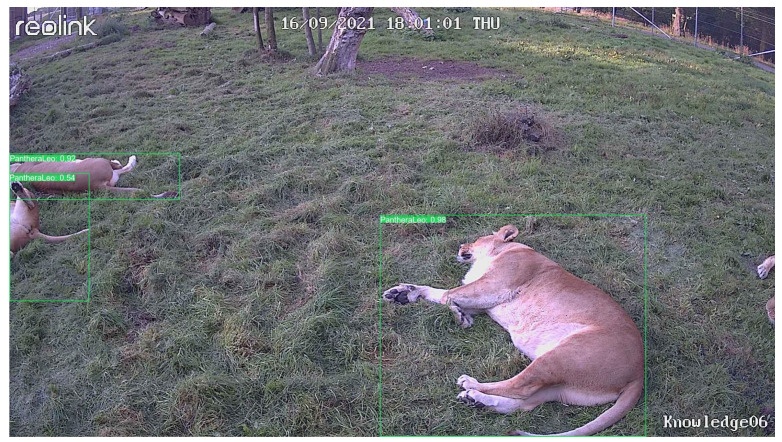
Q7. Based on the species and its habits, what predictions can be made about its activity at the time the camera trap image was taken (e.g., hunting, foraging, resting)?

**Figure A8:**
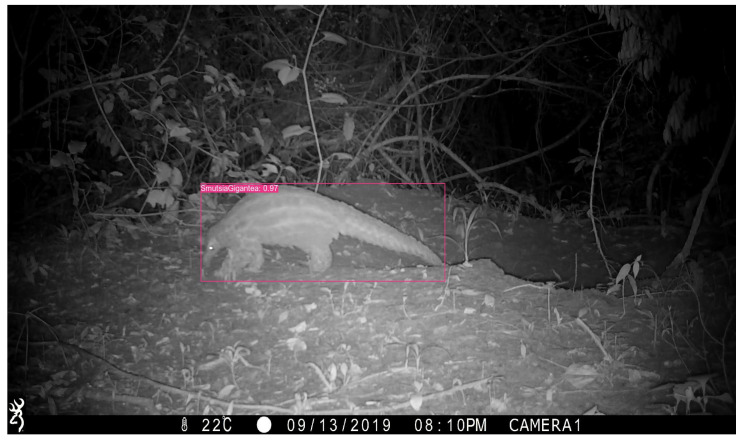
Q8. Read the label on the bounding box around the animal to determine the species. What potential threats, either natural or human-induced, are most relevant to the species in the image, given its current IUCN status and environment?

**Figure A9:**
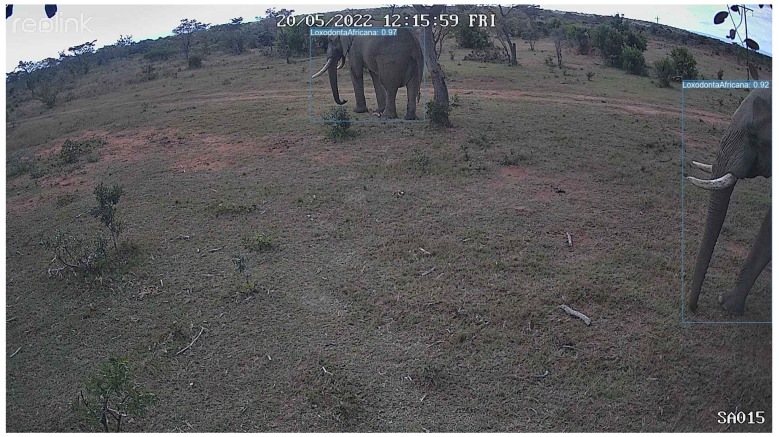
Q9. Read the label on the bounding box around the animal to determine the species. What is the species role in the ecosystem, and how does its presence effect other species or the environment in the area where the image was captured?

**Figure A10:**
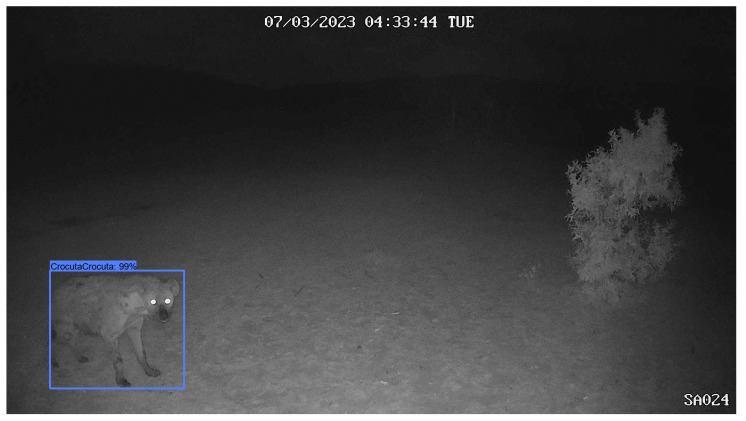
Q10. Read the label on the bounding box around the animal to determine the species. What are the known predators or threats to the species in the image, and are there any visible indicators in the environment that suggest the presence of these threats?

## Data Availability

The data in the study is sensitive and cannot be shared.

## Appendix A

In Appendix A, we include a set of 10 images corresponding to each of the questions evaluated in RAG Q&A Table 2 These images provide visual context for the species and environmental factors discussed in the questions, allowing for a clearer understanding of the challenges and outcomes presented. Each image represents a real-world example of the scenarios encountered in a study, from species identification and environmental observations to the retrieval-augmented generation of supplementary information, like conservation status and ecosystem roles. By including these visuals, we aim to further support the quantitative results with qualitative examples that illustrate the effectiveness and limitations of the proposed system in processing and analysing camera trap images.

## References

[1] O’Connell A.F., Nichols J.D., Karanth K.U. (2011). Camera Traps in Animal Ecology: Methods and Analyses.

[2] Wearn O.R., Glover-Kapfer P. (2019). Snap happy: Camera traps are an effective sampling tool when compared with alternative methods. R. Soc. Open Sci..

[3] Villa A.G., Salazar A., Vargas F. (2017). Towards automatic wild animal monitoring: Identification of animal species in camera-trap images using very deep convolutional neural networks. Ecol. Inform..

[4] Young S., Rode-Margono J., Amin R. (2018). Software to facilitate and streamline camera trap data management: A review. Ecol. Evol..

[5] Nazir S., Kaleem M. (2021). Advances in image acquisition and processing technologies transforming animal ecological studies. Ecol. Inform..

[6] Findlay M.A., Briers R.A., White P.J.C. (2020). Component processes of detection probability in camera-trap studies: Understanding the occurrence of false-negatives. Mammal Res..

[7] Meek P.D., Ballard G., Claridge A., Kays R., Moseby K., O’brien T., O’connell A., Sanderson J., Swann D.E., Tobler M. (2014). Recommended guiding principles for reporting on camera trapping research. Biodivers. Conserv..

[8] Redmon J., Divvala S., Girshick R., Farhadi A. You only look once: Unified, real-time object detection. Proceedings of the IEEE Conference on Computer Vision and Pattern Recognition.

[9] Scotson L., Johnston L.R., Iannarilli F., Wearn O.R., Mohd-Azlan J., Wong W.M., Gray T.N.E., Dinata Y., Suzuki A., Willard C.E. (2017). Best practices and software for the management and sharing of camera trap data for small and large scales studies. Remote. Sens. Ecol. Conserv..

[10] Swanson A., Kosmala M., Lintott C., Simpson R., Smith A., Packer C. (2015). Snapshot Serengeti, high-frequency annotated camera trap images of 40 mammalian species in an African savanna. Sci. Data.

[11] Reynolds J.H., Thompson W.L., Russell B. (2011). Planning for success: Identifying effective and efficient survey designs for monitoring. Biol. Conserv..

[12] Swinnen K.R.R., Reijniers J., Breno M., Leirs H. (2014). A novel method to reduce time investment when processing videos from camera trap studies. PLoS ONE.

[13] Ihaka R., Gentleman R. (1996). R: A language for data analysis and graphics. J. Comput. Graph. Stat..

[14] Beery S., Morris D., Yang S. (2019). Efficient pipeline for camera trap image review. arXiv.

[15] Fennell M., Beirne C., Burton A.C. (2022). Use of object detection in camera trap image identification: Assessing a method to rapidly and accurately classify human and animal detections for research and application in recreation ecology. Glob. Ecol. Conserv..

[16] Zou Z., Chen K., Shi Z., Guo Y., Ye J. (2023). Object detection in 20 years: A survey. Proc. IEEE.

[17] Ehrlich P.R., Wilson E.O. (1991). Biodiversity studies: Science and policy. Science.

[18] Urbano F., Viterbi R., Pedrotti L., Vettorazzo E., Movalli C., Corlatti L. (2024). Enhancing biodiversity conservation and monitoring in protected areas through efficient data management. Environ. Monit. Assess..

[19] Michener W.K., Jones M.B. (2012). Ecoinformatics: Supporting ecology as a data-intensive science. Trends Ecol. Evol..

[20] Zhao Z.-Q., Zheng P., Xu S., Wu X. (2019). Object detection with deep learning: A review. IEEE Trans. Neural Netw. Learn. Syst..

[21] Fergus P., Chalmers C., Longmore S., Wich S. (2024). Harnessing Artificial Intelligence for Wildlife Conservation. arXiv.

[22] Fergus P., Chalmers C., Longmore S., Wich S., Warmenhove C., Swart J., Ngongwane T., Burger A., Ledgard J., Meijaard E. (2023). Empowering wildlife guardians: An equitable digital stewardship and reward system for biodiversity conservation using deep learning and 3/4G camera traps. Remote Sens..

[23] Schneider S., Taylor G.W., Kremer S. Deep learning object detection methods for ecological camera trap data. Proceedings of the 2018 15th Conference on Computer and Robot Vision (CRV).

[24] Lahoz-Monfort J.J., Magrath M.J.L. (2021). A comprehensive overview of technologies for species and habitat monitoring and conservation. BioScience.

[25] Yin S., Fu C., Zhao S., Li K., Sun X., Xu T., Chen E. (2023). A survey on multimodal large language models. arXiv.

[26] Zang Y., Li W., Han J., Zhou K., Loy C.C. (2024). Contextual object detection with multimodal large language models. Int. J. Comput. Vis..

[27] Zhou K., Yang J., Loy C.C., Liu Z. (2022). Learning to prompt for vision-language models. Int. J. Comput. Vis..

[28] Wang H., Li J., Wu H., Hovy E., Sun Y. (2023). Pre-trained language models and their applications. Engineering.

[29] Jain J., Yang J., Shi H. Vcoder: Versatile vision encoders for multimodal large language models. Proceedings of the IEEE/CVF Conference on Computer Vision and Pattern Recognition.

[30] Wang W., Chen Z., Chen X., Wu J., Zhu X., Zeng G., Luo P., Lu T., Zhou J., Qiao Y. Visionllm: Large language model is also an open-ended decoder for vision-centric tasks. Proceedings of the 38th Annual Conference on Neural Information Processing Systems (NIPS 2024).

[31] Sun J., Jacobs D.W. Seeing what is not there: Learning context to determine where objects are missing. Proceedings of the IEEE Conference on Computer Vision and Pattern Recognition.

[32] Lamba A., Cassey P., Segaran R.R., Koh L.P. (2019). Deep learning for environmental conservation. Curr. Biol..

[33] Wang A., Chen H., Liu L., Chen K., Lin Z., Han J., Ding G. (2024). Yolov10: Real-time end-to-end object detection. arXiv.

[34] Micrsoft (2024). Microsoft/Phi-3.5-Vision-Instruct. https://huggingface.co/microsoft/Phi-3.5-vision-instruct.

[35] Vaswani A. Attention is all you need. Proceedings of the 31st Annual Conference on Neural Information Processing Systems 2017.

[36] Lewis P., Perez E., Piktus A., Petroni F., Karpukhin V., Goyal N., Küttler H., Lewis M., Yih W.T., Rocktäschel T. (2020). Retrieval-augmented generation for knowledge-intensive nlp tasks. Adv. Neural Inf. Process. Syst..

[37] Bland L.M., Keith D.A., Miller R.M., Murray N.J., Rodríguez J.P. (2024). Guidelines for the application of IUCN Red List of Ecosystems Categories and Criteria: Version 2.0.

[38] Kleyer M., Bekker R.M., Knevel I.C., Bakker J.P., Thompson K., Sonnenschein M., Poschlod P., van Groenendael J.M., Klimeš L., Klimešová J. (2008). The LEDA Traitbase: A database of life-history traits of the Northwest European flora. J. Ecol..

[39] Gallagher R.V., Falster D.S., Maitner B.S., Salguero-Gómez R., Vandvik V., Pearse W.D., Schneider F.D., Kattge J., Poelen J.H., Madin J.S. (2020). Open Science principles for accelerating trait-based science across the Tree of Life. Nat. Ecol. Evol..

[40] Porras I., Steele P. (2020). Biocredits. A Solution for Protecting Nature and Tackling Poverty Environmental Economics.

[41] Zhang J., Huang J., Jin S., Lu S. (2024). Vision-language models for vision tasks: A survey. IEEE Trans. Pattern Anal. Mach. Intell..

[42] Whytock R.C., Suijten T., van Deursen T., Świeżewski J., Mermiaghe H., Madamba N., Mouckoumou N., Zwerts J.A., Pambo A.F.K., Bahaa-el-din L. (2023). Real-time alerts from AI-enabled camera traps using the Iridium satellite network: A case-study in Gabon, Central Africa. Methods Ecol. Evol..

[43] Vélez J., McShea W., Shamon H., Castiblanco-Camacho P.J., Tabak M.A., Chalmers C., Fergus P., Fieberg J. (2023). An evaluation of platforms for processing camera-trap data using artificial intelligence. Methods Ecol. Evol..

[44] Ma X., Wang L., Yang N., Wei F., Lin J. Fine-tuning llama for multi-stage text retrieval. Proceedings of the 47th International ACM SIGIR Conference on Research and Development in Information Retrieval.

[45] Douze M., Guzhva A., Deng C., Johnson J., Szilvasy G., Mazaré P.E., Lomeli M., Hosseini L., Jégou H. (2024). The faiss library. arXiv.

[46] Padilla R., Netto S.L., Da Silva E.A.B. A survey on performance metrics for object-detection algorithms. Proceedings of the 2020 International Conference on Systems, Signals and Image Processing (IWSSIP).

[47] Wang C.-Y., Liao H.-Y.M., Wu Y.-H., Chen P.-Y., Hsieh J.-W., Yeh I.-H. CSPNet: A new backbone that can enhance learning capability of CNN. Proceedings of the IEEE/CVF Conference on Computer Vision and Pattern Recognition Workshops.

[48] Liu S., Qi L., Qin H., Shi J., Jia J. Path aggregation network for instance segmentation. Proceedings of the IEEE Conference on Computer Vision and Pattern Recognition.

[49] Hosang J., Benenson R., Schiele B. Learning non-maximum suppression. Proceedings of the IEEE Conference on Computer Vision and Pattern Recognition.

[50] Sapkota R., Meng Z., Ahmed D., Churuvija M., Du X., Ma Z., Karkee M. (2024). Comprehensive Performance Evaluation of YOLOv10, YOLOv9 and YOLOv8 on Detecting and Counting Fruitlet in Complex Orchard Environments. arXiv.

[51] Sapkota R., Qureshi R., Flores-Calero M., Badgujar C., Nepal U., Poulose A., Zeno P., Bhanu Prakash Vaddevolu U., Yan P., Karkee M. (2024). Yolov10 to its genesis: A decadal and comprehensive review of the you only look once series. arXiv.

[52] Savard C., Manganelli N., Holzman B., Gray L., Perloff A., Pedro K., Stenson K., Ulmer K. (2024). Optimizing High-Throughput Inference on Graph Neural Networks at Shared Computing Facilities with the NVIDIA Triton Inference Server. Comput. Softw. Big Sci..

[53] Lin T.Y., Maire M., Belongie S., Hays J., Perona P., Ramanan D., Dollár P., Zitnick C.L. Microsoft coco: Common objects in context. Proceedings of the Computer Vision–ECCV 2014: 13th European Conference.

[54] Ren S., He K., Girshick R., Sun J. (2016). Faster R-CNN: Towards real-time object detection with region proposal networks. IEEE Trans. Pattern Anal. Mach. Intell..

[55] LeCun Y., Bengio Y., Hinton G. (2015). Deep learning. Nature.

[56] Abdin M., Aneja J., Awadalla H., Awadallah A., Awan A.A., Bach N., Bahree A., Bakhtiari A., Bao J., Behl H. (2024). Phi-3 technical report: A highly capable language model locally on your phone. arXiv.

[57] Li X., Wang W., Hu X., Yang J. Selective kernel networks. Proceedings of the IEEE/CVF Conference on Computer Vision and Pattern Recognition.

[58] Hussain M. (2024). YOLOv5, YOLOv8 and YOLOv10: The Go-To Detectors for Real-time Vision. arXiv.

[59] Topsakal O., Akinci T.C. Creating large language model applications utilizing langchain: A primer on developing llm apps fast. Proceedings of the International Conference on Applied Engineering and Natural Sciences.

[60] Chen L., Li S., Yan J., Wang H., Gunaratna K., Yadav V., Tang Z., Srinivasan V., Zhou T., Huang H. (2023). Alpagasus: Training a better alpaca with fewer data. arXiv.

[61] Hu T., Zhou X.-H. (2024). Unveiling LLM Evaluation Focused on Metrics: Challenges and Solutions. arXiv.

[62] Pezoa F., Reutter J.L., Suarez F., Ugarte M., Vrgoč D. Foundations of JSON schema. Proceedings of the 25th international conference on World Wide Web.

